# Trend analysis of malaria prevalence in Raya Azebo district, Northern Ethiopia: a retrospective study

**DOI:** 10.1186/s13104-018-4003-4

**Published:** 2018-12-17

**Authors:** Kebede Tesfay, Mokenen Yohannes, Sena Bayisa

**Affiliations:** 1 Department of Medical Laboratory, College of Medicine and Health Science, Adigrat University, Adigrat, Ethiopia; 2 Department of Biomedical, College of Health Science, Mekelle University, Mekelle, Ethiopia

**Keywords:** Ethiopia, Malaria trend, Raya Azebo

## Abstract

**Objective:**

Malaria remains still the leading cause of outpatient visits and death in Ethiopia. However, little is known about its trend in the study area. Hence, this study was aimed to assess 6-year (2011–2016) trend of malaria prevalence. A retrospective cross-sectional study design was conducted to assess 6-year trends of malaria prevalence in Raya Azebo district, North Ethiopia. Malaria case recorded from 2011 to 2016 was extracted using similar format.

**Result:**

A total of 29,930 malaria cases were reported from 2011 to 2016. Of these, 23,018 were confirmed cases while, 6912 were reported as clinical cases. *Plasmodium falciparum* (56.9%) was the most dominated species. Malaria was reported in all age group and both sexes with highest in male and > 15 age categories. The highest peak malaria distribution was occurred in spring season. The overall trends of malaria case were increased in the past 6 years (2011–2016) with exception slightly decreased from 2012 to 2013. Therefore, Strong effort is needed to improve malaria prevention and controlling method in study area.

## Introduction

Malaria is one of the most severe public health problems in tropical regions with high morbidity and mortality rates. The number of malaria cases and deaths was estimated to be 214 million and 438,000 thousands, respectively in 2015 [1]. Most of the cases (88%) and deaths (90%) occurred in the African Region. Most of the affected are children aged less than 5 years, who account for 78% of all deaths. In total, 18 countries account for 90% of infections in sub-Saharan Africa including Ethiopia. During 2000–2013, the scale-up of effective malaria prevention and control interventions saved an estimated 4.2 million lives, with 92% of those being children aged < 5 years, and decreased malaria mortality by 34% in sub-Saharan Africa [2].

Malaria is also a major health problem in Ethiopia, being the leading cause of outpatient visits, admissions, and deaths in the country in 2005–2006 and accounting for over 20% of deaths at all ages [3]. There are estimated 5–6 million malaria cases per year and *Plasmodium falciparum* and *Plasmodium vivax* are the most common species in Ethiopia [4]. Most areas below 2000 m above sea level are considered to be malarious. An estimated 60% of the population lives in areas at risk of malaria transmission. There is marked seasonality in transmission and geographic variation in intensity [5]. 2011 MIS showed that, nationally malaria prevalence was 1.3% in areas below 2000 m; 77% of the positive slides were *Plasmodium falciparum* infections [6].

There has been a substantial increased in coverage of key interventions in the country in the past decade. More than 64 million long lasting insecticidal nets (LLINs) were distributed through mass campaigns between 2005 and 2014 [5]. IRS has also been implemented in many areas. Through the expansion of basic health services, mainly health posts, diagnostic and treatment services have increased over the years. As a result, 2015 world malaria day FMOH report, the number of malaria cases, admissions, and malaria related deaths has been significantly reduced by 67%, 48%, and 55%, respectively [7].

However, the impact of the scale up has not been uniform and the ongoing malaria control interventions are faced with many challenges including vector resistance to insecticides, low coverage of malaria preventive service, poor access to health care, large population movements and limited financial and human resources [8].

Malaria is a major cause of morbidity and mortality in Tigray like the other joining regions. The scale up of malaria control activities in the past decade has resulted in significant reduction in malaria prevalence in Tigray. The proportion of total OPD visits, admissions and deaths due to malaria decreased from 20.5%, 10.5% and 5.1% in 2011/2012 to 11.6%, 4.4% and 1.9% in 2014/15, respectively, in the region [9]. Despite, some reductions in prevalence, malaria remain the second causes of morbidity in 2014/15 with 296,785 (11.55%) cases as outpatient and 5417 inpatient cases [10].

A number of factors influence the epidemiology of malaria in Tigray: Seasonal migration of thousands of laborers to the agricultural areas of the Raya Valley, construction of a railway in progress, increases in agricultural activities in the area. These together are expected to bring changes in infectious malaria diseases, creating a high level of risk to the communities living in the area [11].

Hence, assessing the trend of malaria, in the district is necessary to institute appropriate preventive and control measurement of the disease.

## Main text

### Methods

#### Study area

The study was conducted in Raya Azebo district, Tigray region, Northern Ethiopia. The district is located at northern part, 652 km away from capital Addis Ababa. The *district* had a total population of 135,870, of whom 67,687 were men and 68,183 women; 16,056 or 11.82% are urban inhabitants. Administratively, the district is sub-divided into 20 K*ebeles* lying at an altitude range of 930 to 2300 meters above sea level (masl) [12].

The *district* is characterized by having a bimodal type of rainfall pattern with light rains during the February to April period and heavy rains between July and September. The mean annual rainfall is about 724 mm with mean daily maximum and minimum temperatures of 18.3 °C and 13.93 °C, respectively for the western highlands and 23.44 °C and 19.64 °C, respectively in the valley. Ninety percent of the district is described as “midland” (1500–2300 m) and 10% is considered to be “lowland” (< 1500 m) [13].

#### Study design and period

A retrospective cross sectional study was employed from January 2017 to May 2017. All malaria data was available in the district HMIS health office. This retrospective study was done by reviewing the total malaria case of the district health office for the past 6 years (2011–2016).

#### Source of data

In the study area malaria case patients were treated both clinically and microscopically according the national guideline. Malaria service was given in all health facilities (health post and health center) in the area. In the health post patients are treated clinically and using rapid detection test (malaria antigen detection test).

All health post malaria case data were reported monthly to their nearest health center health management information system office. In addition confirmed malaria cases were reported monthly from laboratory unites to the health center health management information system office. The district haves a total of eight health centers and each have an average of 3 health posts.

Finally all malaria case data was reported monthly from each health center to the district health management information system office (HMIS). Data was stored computerized in HMIS of the district by month, year, sex, clinically treated, RDT and microscopically confirmed cases. This present study was included all malaria case data recorded from 2011 to 2016 in the district health office.

#### Data collection techniques

A similar format was prepared to collect the secondary data from the computer which malaria cases were stored. With the help of the HMIS expert, individual data such as total clinically treated, confirmed case in month and year, types of malaria species and socio-demographic data (age, sex) was collected. Any data such as the socio demographic, malaria diagnosis result which is not properly recorded was excluded.

#### Data analysis

All data was checked for its completeness. Data was analyzed using Microsoft office excel worksheet 2007. The retrospective malaria data was summarized using figure and tables. Specifically the total malaria case in pats 6 year (2011–2016), malaria case by age and sex was summarized using table. On the other hand figure was used to compare specie composition and to assess seasonal distribution of malaria.

### Results

#### Retrospective malaria data

The 6 year (2011–2016) retrospective health facility based malaria data from Raya Azebo *district* is summarized in Table 1. Accordingly, a total of 29,930 malaria cases were reported from the *district* HMIS office. Of these, 23,018 were microscopically and RDT confirmed cases while 6912 were reported as clinical cases. Malaria cases were reported in all years, with the highest number of cases reported in 2016 (28.9%, 8647/29,930). Out of the total microscopically confirmed malaria cases *Plasmodium falciparum* (56.9%), was the most dominated species followed by *Plasmodium vivax* (43.1%).

**Table 1:** Profile of 6-year malaria case report from Raya Azebo district, Tigray, Ethiopia 2011–2016

Year	Microscopically confirmed cases		Total confirmed (%)	Clinical cases (%)	Total (%)
	*P. falciparum* positive (%)	*P. vivax* positive (%)			
2011	575 (35.7)	1036 (64.3)	1611 (7)	2699 (39)	4310 (14.4)
2012	1185 (40.4)	1748 (59.6)	2933 (12.7)	2773 (40.1)	5706 (19.1)
2013	1433 (50.1)	1424 (49.9)	2857 (12.4)	810 (11.7)	3667 (12.2)
2014	1891 (57)	1427 (43)	3318 (14.4)	228 (3.3)	3546 (11.8)
2015	2387 (62.3)	1446 (37.7)	3833 (16.7)	221 (3.2)	4064 (13.6)
2016	5624 (66.4)	2842 (33.6)	8466 (36.8)	181 (2.6)	8647 (28.9)
Total	13,095 (56.9)	9923 (43.1)	23,018 (100)	6912 (100)	29,930 (100)

There was increased malaria case from 2011 to 2012. However, only slightly decreasing trend in number of confirmed malaria cases was observed from 2012 to 2013. This was followed by a steady increase up to 2016. *Plasmodium vivax* malaria showed the same trend—a drop in number of cases between 2012 and 2014 and an upward trend afterwards. On the other hand, *Plasmodium falciparum* cases consistently increased through all years (Table 1).

#### Trends of malaria case in season

**Fig. 1:**
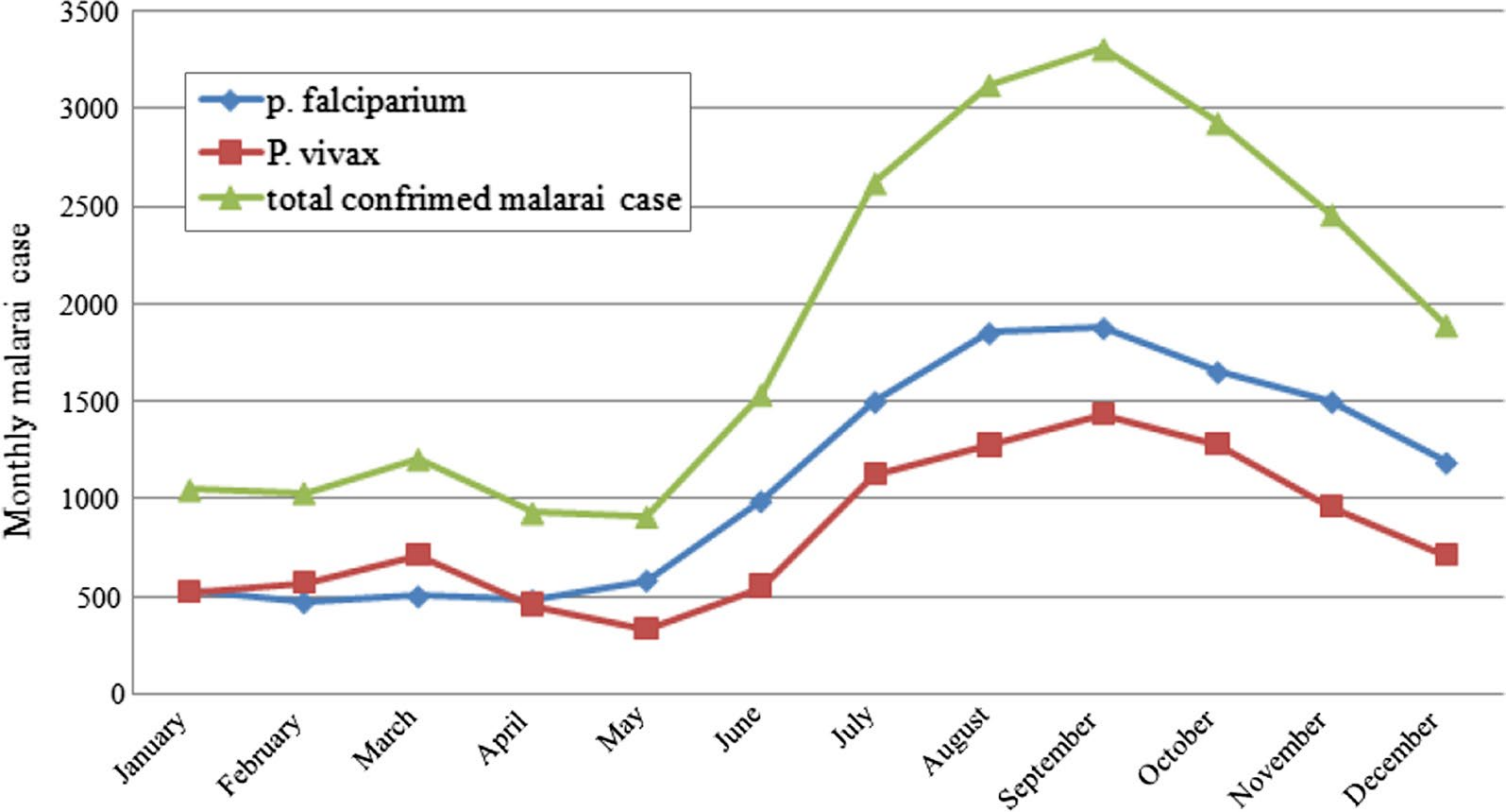
Seasonal distribution of malaria species and total confirmed cases in Raya Azebo *district,* 2011–2016

The seasonal distribution of malaria cases is summarized in Fig. 1. Accordingly, the highest peak occurred in September following the main rainy season (June–August). The smaller peak was observed during the short rainy season (or autumn) in March. Generally, number of malaria cases was lowest during the months of Jan. and Feb, slightly increased in March, dropped a little bit in Apr and May with a steady increase until it reaches its maximum in September with a final drop until reached its minimum in January. *Plasmodium falciparum* dominated almost throughout the year except January to April where it was exceeded by *Plasmodium vivax.*

#### Number of malaria cases by sex and age

Based on the HMIS classification, the total number of confirmed malaria cases reported over the 6 years is summarized by age and sex in Table 2. Accordingly, majority of the affected were males (61.5%) in all age groups compared to females (38.5%). Adults (> 15 years) were the most affected group (55.7%) followed by the 5–14 (29.3%) years old and under five children (15%). The same pattern was exhibited both in males and females.

**Table 2:** Total malaria case by sex and age category in Raya Azebo *district,* 2011–2016

Category of cases	Age and sex								
	Under 5 (%)			5–14 (%)			> 15 (%)		
	M	F	Total	M	F	Total	M	F	Total
Clinical malaria case	612 (53.9)	524 (46.1)	1136	1072 (52.5)	978 (47.5)	2054	2145 (57.5)	1579 (42.6)	3715
*P. falciparum*	899 (56.2)	702 (43.8)	1601	2307 (60.8)	1490 (39.2)	3797	5245 (68.1)	2454 (31.9)	7699
*P. vivax*	979 (56.1)	766 (43.9)	1745	1730 (59.2)	1190 (40.8)	2920	3424 (65.1)	1830 (34.9)	5254
Total (%)	2490 (55.6)	1992 (44.4	4482	5113 (58.3)	3667 (41.7)	8771	10,814 (64.8)	5863 (35.2)	16,677

Age categories	Under-5 (%)	5–14 (%)	> 15 (%)	Total					
Total malaria case	4482 (15)	8771 (29.3)	16,677 (55.7)	29,930					

### Discussion

The present study attempted to assess the trend of malaria prevalence in Raya Azebo *district,* Northern Ethiopia. The general decline in number of clinical malaria cases could be attributed to the provision of diagnostic facilities (notably RDTs) to the health facilities in the district. On the other hand, the nearly stable situation in number of confirmed malaria cases up to 2013 could be due to the scale up of malaria control interventions that started over a decade ago throughout the country. However, the upward trend that followed over the years reaching a peak in 2016 is of real concern and could be due to a number of factors such as, global climatic changes like (irregular rain-full, increased temperature) and increased modernized agricultural activities like irrigation, construction many of micro dams, and ponds in the study area. In addition, ongoing construction of new railway cross the study area becomes a factor for easily breeding of mosquitoes.

*Plasmodium falciparum* was observed consistently increase throughout all 6 years; This might be due to inconsistency malaria preventing methods in the areas and increasing the drug resistance behavior of *Plasmodium falciparum. Plasmodium vivax* case was observed slightly decreased from 2012 to 2014 but it increased up to 2016, this could be due to poor malaria preventing in the area. The distribution of *Plasmodium falciparum* (56.9%), *Plasmodium vivax* (43.1%) in this study was slightly similar with the study of around Gilgel-Gibe Hydroelectric Dam (GGHD), which accounts *Plasmodium falciparum* (54.6%) and *Plasmodium vivax* (41.6%) [14].

Malaria case occurs in all month and years in this study area. The highest peak malaria case was observed during in September month (spring season). This peak malaria season in study area was similar with many studies in the country [15–19]. The second peak malaria case was observed on summer (June to August) season similar with study of [14, 20] this might probably due to relapsing behavior of some malaria parasite and irregular rain-full in the area. Probably many factors might be influenced the seasonal distribution of malaria, e.g. climatic variables, behavioral and biological variation across mosquito species, environmental factors and economic factors.

Regarding the age groups, > 15 years (55.7%) were highly affected age groups followed by 5–14 (29.3%) years old and under five children (15%). The prevalence of malaria parasites among males (61.5%) was higher than females (38.5%). This could be due to male was more responsible to control the environmental activities than female like participated in agricultural activities, long traveling habitat to other sites.

### Conclusion

In this present study the overall trends of total confirmed malaria case were increased in the past 6 years (2011–2016) with exception slightly decreased a case from 2012 to 2013. Therefore, malaria prevention and controlling method should be strengthened in the study area.

## Limitation

Data on all febrile cases examined for malaria parasites was not available in the *woreda* health management information system data base. Only those clinical and lab confirmed positive cases were reported. Due to this it was not possible to calculate the percentage of febrile cases who were positive for malaria. The health management information system data appeared to be incomplete.

## Abbreviations

ITN: insecticide treated net
HMIS: health management information system
LLIN: long lasting insecticidal nets
FMOH: federal ministry of health
RDT: rapid detection test
MIS: malaria indictor survey
WHO: World Health Organization
